# Survival and Cardioprotective Benefits of Long-Term Blueberry Enriched Diet in Dilated Cardiomyopathy Following Myocardial Infarction in Rats

**DOI:** 10.1371/journal.pone.0007975

**Published:** 2009-11-19

**Authors:** Ismayil Ahmet, Edward Spangler, Barbara Shukitt-Hale, James A. Joseph, Donald K. Ingram, Mark Talan

**Affiliations:** 1 Laboratory of Cardiovascular Science and Intramural Research Program, National Institute on Aging, National Institutes of Health, Baltimore, Maryland, United States of America; 2 Laboratory of Experimental Gerontology, Intramural Research Program, National Institute on Aging, National Institutes of Health, Baltimore, Maryland, United States of America; 3 United States Department of Agriculture-Agricultural Research Service (USDA-ARS), Human Nutrition Research Center on Aging, Tufts University, Boston, Massachusetts, United States of America; 4 Nutritional Neuroscience and Aging Laboratory, Pennington Biomedical Research Center, Louisiana State University System, Baton Rouge, Louisiana, United States of America; University of Washington, United States of America

## Abstract

**Background:**

Despite remarkable progress in treatment of chronic heart failure (CHF) over the last two decades, mortality, personal suffering and cost remain staggering, and effective interventions are still a challenge. Previously we reported that a blueberry-enriched diet (BD) attenuated necroapoptosis and inflammation in periinfarct area in a rat model of myocardial infarction (MI).

**Objectives:**

To test the hypothesis that BD will attenuate the course of CHF, including mortality and cardiac remodeling during the first year after induction of MI in rats.

**Method and Results:**

Two weeks after coronary artery ligation, rats were divided into two groups of similar average MI size, measured by echocardiography, and then12-mo dietary regimens were initiated as follows: ad libitum regular diet (control, CD, n = 27) and isocaloric food with 2% blueberry supplement (BD, n = 27) also available ad libitum. These dietary groups were compared to each other and to sham group (SH). Mortality over the 12 mo was reduced by 22% in BD compared with CD (p<0.01). In the course of developing CHF, BD had no effect on the body weight, heart rate or blood pressure. Bi-monthly Echo revealed significant attenuation of the LV chamber remodeling, LV posterior wall thinning, and MI expansion in BD compared with CD. In fact, BD arrested the MI expansion.

**Conclusion:**

This is the first experimental evidence that a blueberry-enriched diet has positive effects on the course of CHF and thus warrants consideration for clinical evaluation.

## Introduction

Chronic heart failure (CHF) is a wide spread, highly disabling condition usually occurring subsequently to ischemic heart disease and myocardial infarction, however hypertension, valvular disease and cardiomyopathy are also contributing etiologies [1]. In developing countries, an estimated 2–3% of the adult population and almost 10% of those over 65years of age suffer from this condition. Moreover, in the age cohort of 70–80 years, the prevalence of CHF reaches 20% [2], [3]. For instance, in 2004, 12.5% of death certificates issued in the US mentioned CHF [4]. The estimated annual cost of health care attributed to CHF exceeds $35 billion in the US, and in the United Kingdom the cost equals 2% of the total budget of the National Health Service [3], [4].

Remarkable progress has been achieved over the last two decades in treatment of CHF. The use of beta adrenoreceptor blockers, angiotensin-converting-enzyme inhibitors, aldosterone antagonists, and resychronization therapy revolutionized the management of CHF [5]. However, despite recognized successes, the overall annual mortality associated with CHF remains around 10% [5], [6], and quality of life among survivors is dramatically reduced as the disease progresses [7], [8]. Thus, a search for new therapeutic interventions to improve the course of CHF continues.

Reports over the last several years suggested that, at least in animal experiments, supplementation of the regular diet with blueberry produces a tissue protective effect: blueberry extract and blueberry-enriched diets have been shown to attenuate age-related behavioral and neuronal deficits [9]–[12], to inhibit inflammatory cytokines in rat glial cells [13], and even to reduce hippocampal cell loss following experimentally induced stroke [14]. We have recently reported that myocardial infarct (MI) size, as well as necroapoptosis and inflammation in peri-infarct area at 24 hrs after MI induction, are significantly reduced in rats maintained on blueberry-enriched diet (BD) prior to induction of MI in comparison with rats on the control diet (CD) [15]. Our data also suggested that initiation of BD after MI induction might attenuate left ventricular (LV) remodeling and MI expansion.

It is generally accepted that a major factor leading to progression of CHF is cumulative cell loss in myocardium [16]. Since multiple reports suggested that BD possesses tissue-protective (antiapoptotic and anti-inflammatory) properties, the objective of this study was to test the effectiveness of 12-mo long blueberry-enriched diet in the rat model of post MI dilated cardiomyopathy with mortality as a primary outcome.

## Methods

### Experimental Design

Male Wistar rats (Charles River Laboratories Inc., Wilmington, MA), weighing 225–280g, were housed and studied in conformance with the NIH Guide for the Care and Use of Laboratory Animals, Manual 3040-2 (1999), with institutional Animal Care and Use Committee approval. Rats were maintained on ad libitum food (NIH-07 Mouse/Rat diet) with permanent access to filtered water. The left descending coronary artery was ligated in 86 rats. An additional 10 rats underwent a sham operation (SH) without actual coronary ligation. Two weeks after surgery, LV dimensions, ejection fraction (EF) and MI size were measured by echocardiography (Echo) in surviving coronary ligated and SH rats. Animals with an MI size greater than 20% but less than 50% of LV were divided into 2 groups of similar MI size (average and variability). One MI group, as well as SH (n = 10), continued to receive a regular, control diet (CD, n = 27), while for another MI group the regular diet was replaced with a blueberry-enriched diet (Harlan Teklad Madison, WI), (BD, n = 27), also available ad libitum as described previously [15]. Briefly, the blueberry enriched diet was isocoloric with control diet, but contained 2% of blueberry extract. New dietary regimens started 2 weeks after coronary ligation and continued for 12 months (4 weeks were counted as 1 month). Animals were inspected daily for signs of moribundity by a person blinded to dietary assignments. Moribund animals were euthanized, and their hearts were harvested for MI measurements. Daily records of dead or euthanized animals were used to calculate continuous mortality curves. Echo was repeated bi-monthly following the initiation of treatment. Body weight and blood pressure (non-invasive tail-cuff technique, Coda-6, Kent Scientific, Torrington, CT) were measured at the same time as Echo. Following the final Echo all surviving rats were euthanized and their hearts harvested for histological evaluation.

### Coronary Artery Ligation

Rats were anaesthetized with Isoflorene (2% in Oxygen). The surgical procedure was performed as previously described [17].

### Echocardiography

Echocardiography (Sonos 5500, a 12 MHz transducer) was conducted under light anaesthesia by sodium pentobarbital (30 mg/kg, i.p.) as previously described [18]. Briefly, parasternal long axis views were obtained and recorded to ensure that the mitral and aortic valves and the apex were visualized. Short axis views were recorded at the mid-papillary muscle level. Endocardial area tracings, using the leading edge method, were performed in 2D mode (short and long axis views) from digital images captured on cineloop to calculate end-diastolic and end-systolic LV areas. End-diastolic volume (EDV) and end-systolic volume (ESV) were calculated by a modified Simpson's method. Ejection fraction (EF) was then derived as EF = (EDV−ESV)/EDV×100. Left ventricular mass (LVM) was calculated from 2D mode. The MI size at the mid-papillary muscle level was estimated from 2D short axis LV images and expressed as a percent of the LV endocardial circumference. Infarct area was identified as a sharply demarcated section of the LV free wall that failed to thicken during systole. The length of the akinetic part of the LV endocardial circumference was measured from freeze-frame images at end-diastole. Posterior wall thickness (PWth) was measured from M-mode. All measurements were made by a single observer, who was blinded to the identity of the tracings, and were averaged over three to five consecutive cardiac cycles. The reproducibility of measurements was assessed in two sets of baseline measurements in 10 randomly selected rats, and the repeated measure variability did not exceed ±5%.

### Histological Acquisition

Histological staining and analyses were performed as previously described [18]. Briefly, the hearts were isolated and weighed. Myocardial segments from the mid-papillary muscle level were imbedded in the paraffin, sectioned (5µm) and stained with Masson's trichrome and hematoxylin - eosin staining (H&E). MI size was expressed as an average percentage of the LV endocardial and epicardial circumferences that were identified as infarct in the Masson's trichrome staining sections.

### Statistical Analyses

All data are expressed as mean±SEM. Mortality was reported via Kaplan-Meier survival analyis. Differences among survival curves were assessed using Logrank statistical analyses (GraphPad Prism, 4.02). Reported Echo indices were analyzed using the repeated measures Linear Mixed-Effects model. Each response variable was analyzed for main effects of group and time as well as their interaction. If the group-time interactions were statistically significantly different among groups, comparisons at different time point were conducted and their outcome was Bonferroni corrected for multiple comparison. The model was fit using proc mixed in SAS 9.1. Power Analysis (PASS 2008 - Repeated Measures ANOVA) indicated that, for the main effect, a sample size exceeding 10 animals per group would correspond to a power above 80%; for the interaction effect, the power for all variables would be above 70% for samples of 15 or larger. Group differences in histological data were assessed by Student's t-test. Statistical significance was assumed as p<0.05.

## Results

### Early Mortality Following Coronary Ligation and Treatment Assignment

Coronary ligation was performed on a total of 86 rats and a sham operation on 10 rats. During the first 24 hrs after surgery 15 animals died, and 7 more rats died during the first 2 weeks following surgery. There was no mortality among the 10 sham operated rats. Two weeks following surgery the 64 rats surviving coronary ligation and 10 sham operated rats underwent echocardiography, at which time their pretreatment (baseline) MI size, LV volumes, and EF were determined. Ten rats, in which MI size was less than 20% or more than 50% of LV, were excluded from the therapeutic interventions. Fifty four rats with an average MI size of 31±1.5% of LV were divided into two dietary groups (n = 27 in each group), that did not differ with respect to their average baseline echo-derived LV morphometric parameters (EDV and ESV), EF and MI size. The 10 sham operated rats served as a control and remained on a regular diet.

Table 1 lists the echo derived **pretreatment** EDV, ESV and EF, PWth and MI size for the two experimental groups compared to the sham group two weeks after surgery. Early, **pretreatment**, LV remodeling was similar in both coronary ligated groups and consisted of substantial increases in EDV (164–171% ) and ESV (397–430%), and a 57–62% decline of EF compared to SH. The thickness of posterior wall in coronary ligated rats did not vary significantly from SH at this time. All measured Echo indices and MI size were similar between two coronary ligated groups.

**Table 1:** Echo derived pre-diet indices of LV remodeling and MI size at 2 weeks following coronary ligation or sham operation.

	SH	CD	BD
EDV (µL)	227±6	616±14***	606±19***
ESV (µL)	90±5	466±17***	464±22***
PWth (mm)	1.51±0.04	1.45±0.06	1.35±0.05
EF(%)	60.7±1.8	24.9±1.4***	22.5±1.4***
MI(%)	NA	30.1±1.3	31±1.5

*** - p<0.001 vs SH.

### Mortality

**Figure 1:**
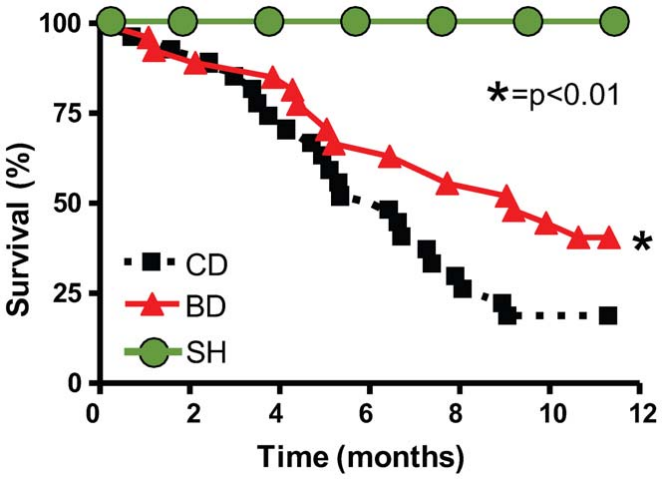
12-mo survival after MI. Kaplan-Meier survival curves following induction of MI in a sham operated group (SH- n = 10), and two groups with MI maintained on control diet (CD - n = 27) or blueberry-enriched diet (BD - n = 27).

Figure 1 illustrates the Kaplan-Meier survival curves for the two groups of experimental animals maintained on different dietary regimens and the sham operated group during one year following initiation of treatment. No animals from SH died during the observation period. In the MI group on control diet (CD) mortality reached 50% at 6 mo and 81% at 12 mos. The positive effect of BD treatment on survival became statistically significant by the 7th month and continued to increase with time so that by the end of the year the survival among BD improved by 22%, compared to the CD group (p<0.01, Logrank test).

### Body weight and hemodynamics

**Figure 2:**
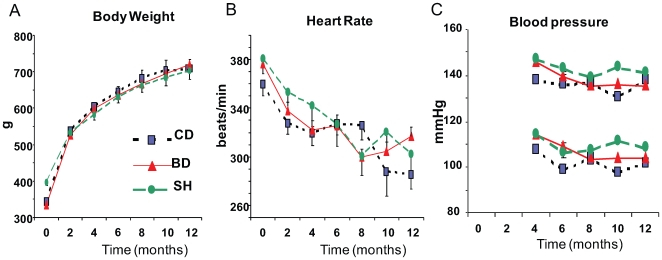
Effect of blueberry-enriched diet on basic physiological parameters. Body weight (A), heart rate (B), and arterial blood pressure (C). Note, results of blood pressure measurements are not available for the first 2 time points.

At the baseline, i.e., 2 weeks after surgery, mean body weight was slightly lower in MI animals (BD and CD) than in SH (Fig. 2A). However, starting at the second month, the body weight was similar among all three groups. The BD did not affect the rate of body growth. Heart rate (Fig. 2B) steadily declined as animals became older and bigger, however there were no differences among groups, i.e., neither surgery, nor the diet, affected the age-related reduction in heart rate. Results of non-invasive measurements of blood pressure are presented in Figure 2C. Systolic and diastolic blood pressures did not differ between diet groups and both diet groups did not differ from SH.

### Infarct Expansion

**Figure 3:**
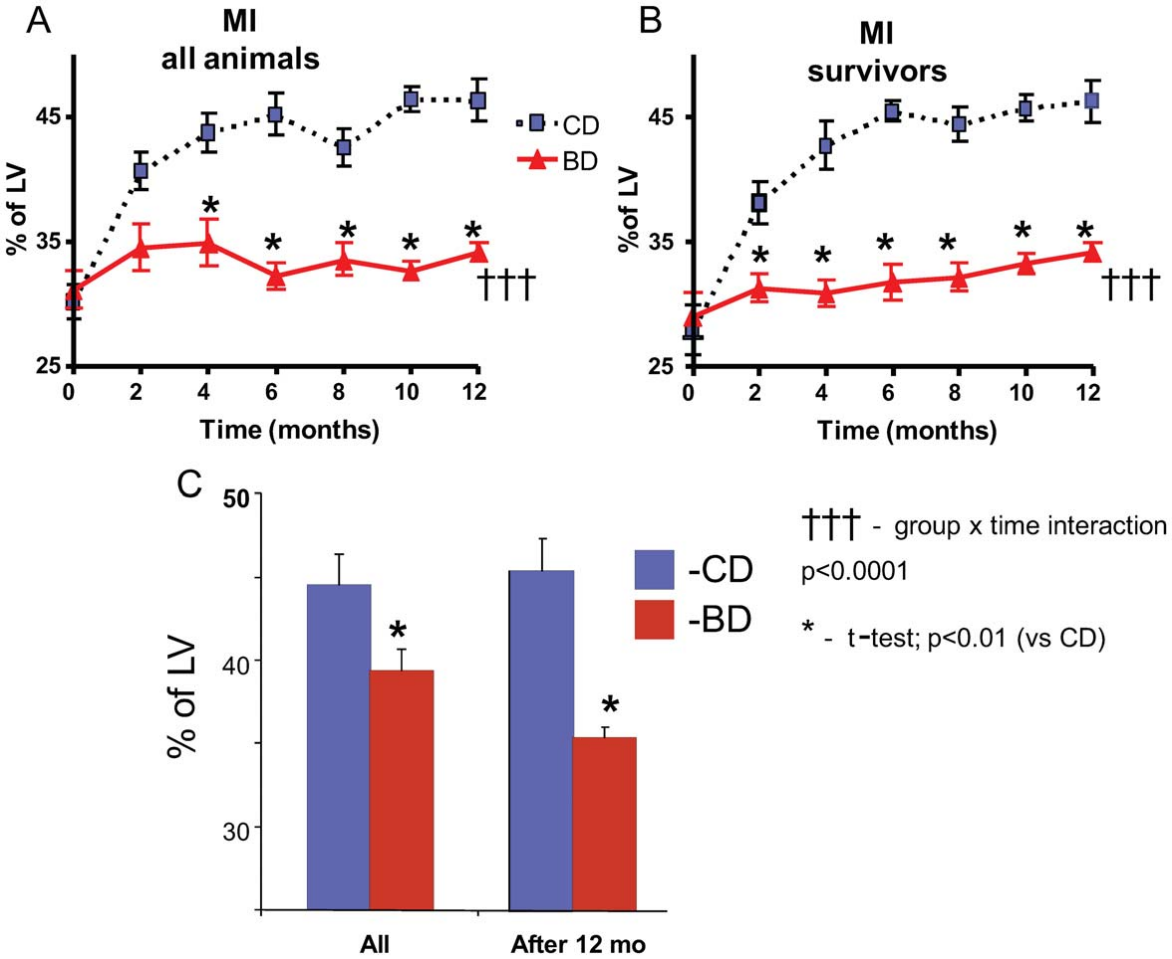
Effect of blueberry-enriched diet on MI expansion. MI expansion was measured by echocardiography (A, B) and histologically (C) in all animals and in animals that survived 12 months following induction of MI.

Figure 3 illustrates the MI expansion during 12 months of treatment. Fig. 3A presents data for all animals, while Fig. 3B illustrates the results obtained only from the rats that survived until the end of the study. In both panels A and B the MI expansion was assessed bi-monthly from Echo measurements and expressed as the percent of LV perimeter. Figure 3C illustrates the MI size, assessed from histological preparations at the termination of the study, or at the time of death. Echo derived and histological measurements were highly correlated (R^2^ = 0.7). In CD rats the average MI expanded from 30.1±1.3% of LV 2 weeks after coronary ligation to 46.2±1.7% of LV at the end of 12 months, i.e., during the 12 months of observation the MI expanded by 50%. MI expansion was attenuated in BD (p<0.001 vs CD). In fact, the average MI size in BD never exceeded the baseline level, and was significantly smaller than in CD at all time points except baseline, at which time the MI was similar between groups. MI size measured histologically in rats that survived 12 month after coronary ligation also indicated a significant attenuation of MI expansion in BD (35% of LV vs 45% in CD, p<0.01).

### LV Remodeling and Function

**Figure 4:**
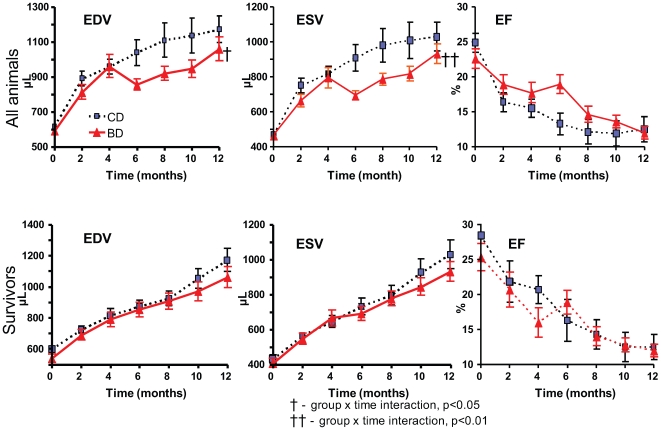
Effect of blueberry-enriched diet on cardiac remodeling. LV end-diastolic volume (left panels), end-systolic volume (middle panels), and ejection fraction (right panels) estimated by bi-monthly Echo during 12 months following induction of MI in rats maintained on control or blueberry-enriched diets. Upper panels - all animals. Lower panels - only animals that survived 12 months following induction of MI.

Figure 4 illustrates the progression of LV remodeling (EDV and ESV expansion) and functional decline (EF reduction) during the experimental period. Upper panels represent average measurements from all animals at each time point, while lower panels represent only animals that survived to the end of the study. In CD animals the LV volume monotonically increased by 90% and 120% for EDV and ESV respectively, while EF declined by 50%. At the end of the observation period EDV in CD animals averaged 1170±75 µL, ESV was 1028±83 µL, and EF was 12.5±1.8%. For comparison (data not shown) the LV EDV in SH animals at the end of observation was only 506±13 µL, ESV was 242±10 µL, and EF was 52±1.9%. The expansion of LV volumes was significantly attenuated in BD compared to CD when all animals (upper panels) were compared (group×time interaction derived from repeated measures ANOVA was p<0.05 and p<0.01 for EDV and ESV respectively). The differences between diet groups became noticeable starting around 6 months. However, the LV expansion was similar in both diet groups when only survivors were compared (lower panels). During the 12 months of the study the decline in EF was not significantly attenuated in BD, compared to CD, with exception of slight improvement in BD at the middle of observation period.

**Figure 5:**
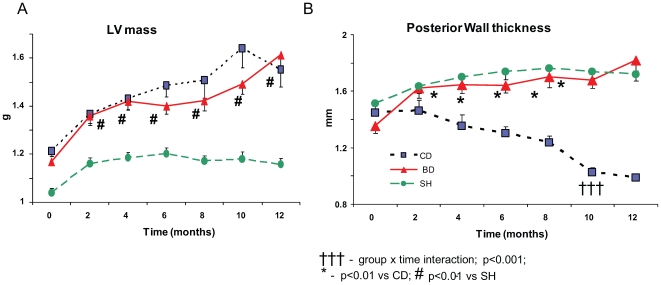
Effect of blueberry-enriched diet on cardiac remodeling. LV mass (A) and posterior wall thickness (B) estimated by bi-monthly Echo during 12 months following induction of MI in rats maintained on control or blueberry-enriched diets and in sham operated rats.

Echo calculated LVM (Figure 5A) increased in both MI groups compared to SH, and was not affected by diet. On the other hand, posterior wall thickness (Figure 5B) became significantly reduced in MI animals maintained on control diet compared to SH, and did not differ from SH in MI animals fed the BD.

## Discussion

In our previous study we have demonstrated for the first time that a natural product such as blueberry, supplemented to a regular diet so that to constitute about 2% of daily food intake, protected the myocardium of rats from ischemic damage induced by ligation of a coronary artery via reducing necro-apoptosis in periinfarct area and ultimately reducing the size of developing myocardial infarct. We have also demonstrated that isolated cardiomyocytes from rats maintained on the blueberry-enriched diet have increased mitochondrial permeability threshold - a finding that provides an apparent mechanistic basis for developed cardioprotection. The present study extended previous findings and proved in a one-year long experiment on rats that BD, initiated two weeks after induced myocardial infarction, attenuated LV remodeling and MI expansion, prevented the progressive thinning of LV wall, and actually reduced by 22% the mortality associated with developing chronic heart failure.

It is generally accepted now that chronic heart failure is accompanied by low, but persistent, level of cordiomyocyte death [19]. It had been shown that at any given time approximately 0.25% of myocytes undergo an apoptotic transformation in the failing human heart. This rate of apoptosis is 100-fold more than in normal heart and more than enough to ensure the progression of failure. For instance it was shown in a mouse model that even a very small increase of the rate of cardiomyocyte loss (0.023% compared to a normal rate of 0.002%) is sufficient to cause a development over time of lethal dilated cardiomyopathy [20]. The rate of cardiamyocyte necrosis is also elevated in the failing human heart to a degree exceeding the rate of apoptosis [21]. Increased rate of apoptosis and necrosis in the failing heart presents a valuable therapeutic target, and benefit of pharmacologically suppressed apoptosis had been demonstrated in experimental models [22].

It is reasonable to assume that an antiapoptotic effect of the blueberry-enriched diet that we demonstrated in the acute stage of experimental myocardial infarction [15] is responsible for amelioration of cardiac remodeling and MI expansion, and ultimately reduced mortality in developing post-MI chronic heart failure as we observed in the present study. It is certain that increased mitochondrial permeability threshold associated with prolonged blueberry-enriched diet in rats [15] plays an important role in cardiac protection in the present, post-MI dilated cardiomyopathy model. As to the exact nature of increased mitochondrial permeability threshold, the possibilities are too numerous, such that their identification, while important and possible, is out of scope of the present work. For instance, the antioxidant properties of blueberries are well known [23] and ROS scavenging mechanisms were identified as one of the mechanisms involved in the increase of mitochondrial permeability threshold [24]. On the other hand there are numerous and not necessarily antioxidant mechanisms which could be involved. For example, a large number of kinase-signaling cascades had been shown to be involved in regulation of the mitochondrial permeability threshold, for instance PKA, PKB/Akt, p70S6K, PKC, PKG, and GSK-3β [25].

In summary, we present the first experimental evidence that in a rat model of post myocardial infarction dilated cardiomyopathy, a blueberry-enriched diet improved one-year survival, attenuated LV remodeling, prevented LV wall thinning, and arrested MI expansion.
